# The American Journal of Medical Sciences

**Published:** 1847-08

**Authors:** 


ARTICLE XV.

*The American Journal of the Medical Sciences.* Edited by ISAAC HAYS, M.D., Surgeon to Will's Hospital, &c. April 1847. (In Exchange.)

The American Journal of the Medical Sciences may, without disparagement to any other, be said to stand at the head of American Medical Journals, being the oldest and certainly one of the very best. Under the head of original communications it contains articles from eminent members of the profession in every part of this country. Its reviews and bibliographical notices are numerous and impartial, and under the heads of Foreign and Domestic Summaries it sums up with great care whatever is new and valuable in periodical medical literature. The tone of the work, from its first commencement to the present time, has been uniformly elevated, dignified and courteous. In this respect we think its influence has been highly favourable upon the entire profession. We would recommend it to any physician. D. B.

